# Prolonged postoperative ileus in gastric surgery: Is there any difference between laparoscopic and open surgery?

**DOI:** 10.1002/cam4.2459

**Published:** 2019-08-05

**Authors:** Wenquan Liang, Jiyang Li, Wang Zhang, Jie Liu, Mingsen Li, Yunhe Gao, Ning Wang, Jianxin Cui, Kecheng Zhang, Hongqing Xi, Bo Wei, Lin Chen

**Affiliations:** ^1^ Department of General Surgery The First Medical Center of Chinese People's Liberation Army General Hospital Beijing People's Republic of China; ^2^ General Surgery Institute The First Medical Center of Chinese People's Liberation Army General Hospital Beijing People's Republic of China; ^3^ Department of Vascular and Endovascular Surgery The First Medical Center of Chinese PLA General Hospital Beijing China; ^4^ Anorectal Desease Diagnosis and Treatment Center Tianjin Union Medical Center Nankai University Affiliated Hospital Tianjin China

**Keywords:** gastric surgery, laparoscopic surgery, open surgery, paralytic ileus, prolonged ileus

## Abstract

**Background:**

Prolonged postoperative ileus (PPOI) is a common complication after abdominal surgery, but data about risk factors of PPOI for patients with gastric cancer are rare. We sought to investigate the impact of laparoscopic versus open surgery for PPOI after gastric cancer surgery.

**Methods:**

A retrospective cohort study was conducted using a registry database consecutively collected from June 2016 to March 2017. PPOI was defined as no bowel function persisting for more than 4 days. Univariate analysis and multiple logistic regression models were performed to investigate risk factors, and stratified analysis was carried out to examine the primary association at different levels of a potential confounding factor.

**Results:**

A total of 162 patients composed of 63 patients undergoing laparotomy and 99 patients undergoing laparoscopy were enrolled and PPOI was observed in 32 (19.75%) patients. Risk factors significantly correlated with PPOI were as follows: open surgery, older age, late surgical pathologic staging, postoperative use of opioid analgesic, low level of postoperative albumin and serum potassium. Compared to open surgery, the laparoscopic surgery was a strong protective factor for PPOI after adjusting related variables (OR = 0.17, CI: 0.05‐0.52, *P* = .002). There was an interaction between surgical methods and the postoperative WBC level (*P* for interaction = .007). In the two group stratified analysis of WBC, laparoscopic surgery had a significant lower risk of PPOI than open group for the patients with WBC counts above the middle level in crude or adjusted models. This result remained significantly in the three group stratified analysis for the patients with WBC counts in the middle and or high tertile groups.

**Conclusions:**

PPOI is a common postoperative complication of patients after gastrectomy. Laparoscopic surgery is associated with decreased risk of PPOI in gastric surgery. Patients who underwent open surgery and presented with high level of WBC should be cautious with PPOI.

## Graphical abstract

Stratified analyses of surgical approaches on PPOI showed that there was an interaction between surgical approaches and the subgroup of postoperative WBC.

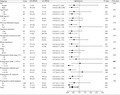

## 1 INTRODUCTION

Postoperative ileus (POI) is a phase of a symptomatic delay of gastrointestinal motility after abdominal surgery. The clinical manifestations include abdominal pain, nausea, vomiting, moderate to severe sick, intolerable of a solid diet and a delayed passage of flatus and stool, which usually resolves spontaneously within 2 to 3 days.^1^, ^2^ If the symptoms persist for more than 4 days, prolonged postoperative ileus (PPOI) is defined.^2^ PPOI was an essential contributor to the prolonged hospitalization and readmission, increasing substantial physician workload and high costs on the healthcare system.^3^ The incidence of PPOI was reported to vary from 3% to 32%, and there were some differences among the sites of abdominal surgery.^4^, ^5^, ^6^ However, a majority of the previous studies on PPOI were based on patients referred to colonic or rectal resection, and little data existed on gastrectomy.^7^, ^8^

Gastric cancer is the third leading cause of cancer death worldwide although its incidence is decreasing.^9^ Management of standard surgical approach remains controversial. Laparoscopic gastrectomy has gained popularity in the treatment of early‐stage gastric cancer.^10^ However, open surgery is still the current standard for advanced gastric cancer according to the Japanese gastric cancer treatment guidelines.^11^ A recent randomized clinical trial compared laparoscopic distal gastrectomy with open distal gastrectomy and did not result in inferior disease‐free survival at 3 years among patients with locally advanced gastric cancer.^12^ The operational advantage of laparoscopic surgery on gastrectomy has been identified, including decreased blood loss, shorter time to ambulation and faster recovery of bowel function, leading to a reduced rate of PPOI. However, anastomotic leakage and pneumoperitoneum‐related complications seemed more likely to occur after using the laparoscopic approach,^11^, ^13^ leading to an increased rate of PPOI. To our knowledge, no study to date focused explicitly on the impact of laparoscopic vs open surgery for PPOI after gastric cancer surgery. Thus, the present study aimed to compare the effects of different surgical approaches on PPOI in a cohort of consecutive patients who had undergone a gastrectomy.

## 2 PATIENTS AND METHODS

### 2.1 Study population

**Figure 1:**
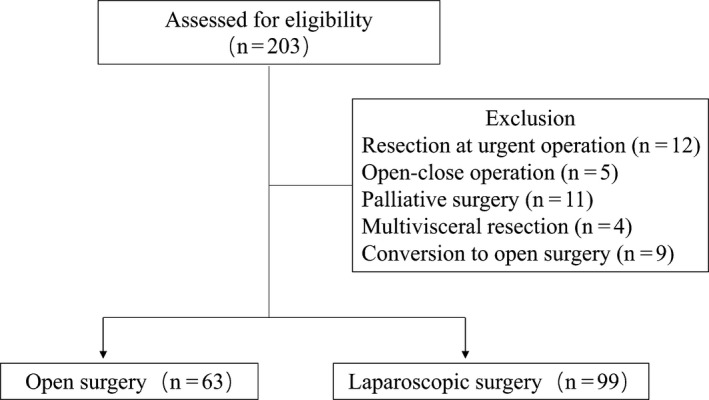
Patient enrollment flowchart

A retrospective cohort study was carried out using a PPOI registry database consecutively collected between June 2016 and March 2017 in Chinese PLA General Hospital. Patients diagnosed with resectable gastric cancer assessed by endoscopic examination and radiographic assessments were enrolled in this study. Surgical approaches were confined to the open technique and the laparoscopic technique. The decision to use either approach was based on the doctor‐patient preoperative communication. The inclusion criteria were as follows: diagnosed with resectable gastric cancer, age ranged from 18 to 80 years, well‐tolerated with the surgical procedure, with American Society of Anesthesiologists classification status I to II, able to give informed written consent. Participants were excluded based on the following criteria: resection at urgent operation, open‐close operation, palliation surgery, multi‐visceral resection, planned laparoscopic surgery converted to open surgery, the presence of a mental disorder, pregnancy. A total of 203 patients who underwent gastric surgery were assessed for potential enrollment in the study (Figure 1). Forty‐one patients were removed from this study, who fulfilled the exclusion criteria including resection at urgent operation (n = 12), open‐close operation (n = 5), palliation surgery (n = 11), multi‐visceral resection (n = 4), planned laparoscopic surgery converted to open surgery (n = 9). Ultimately, a total of 162 patients composed of 63 patients undergoing laparotomy and 99 patients undergoing laparoscopy were left for analysis.

### 2.2 Diagnosis of PPOI

The definition of PPOI was adopted from the results of a systematic review and global survey.^2^ The validity of this concept was universally accepted by a variety of investigators.^5^, ^7^, ^14^, ^15^, ^16^, ^17^ Accordingly, diagnoses of PPOI were identified if two or more of following events after day 4 postoperatively: (a), nausea or vomiting; (b) inability to tolerate an oral diet over the prior 24 hour; (c) absence of flatus over the prior 24 hours; (d) abdominal distension; (e) radiologic confirmation.^2^

### 2.3 Data collection procedures

Data were prospectively collected before the assessment of PPOI, and this study was designed after complementing data entry, which avoided the interference of data collection from investigators. Patient background factors, including sex, age, body mass index, and previous abdominal surgery were collected on admission. Features of the operation consisted of operative time, estimated operative blood loss, perioperative transfusion and type of operation. All operations including open resections and laparoscopic‐assisted procedures were included in the study. Postoperative physical examination, normal hematopoietic and biochemical tests were examined on the first day after surgery. Markers of inflammation, including postoperative body temperature and leukocyte, were collected. Postoperative albumin was also included, which was considered as a surrogate marker for nutritional status and as a negative inflammatory marker. Postoperative potassium level was reported to be associated with the development of prolonged ileus and was recorded in the study by Kuruba.^18^ Pathologic staging was classified according to the seventh edition of the AJCC TNM staging system of gastric cancer. Whether opioid analgesics were used after surgery was assessed by the medical‐therapy group's evaluation of postoperative pain which was collected in the study.

### 2.4 Statistical analysis

Data were reported as a mean ± standerd deviation for continuous variables and as a patient percentage for categorical variables. The differences between groups were calculated by using One‐Way ANOVAs, Kruskal‐Wallis H tests, and Chi‐squared tests as appropriate. Univariate and logistic regression analyses of factors associated with PPOI were performed. The non‐adjusted, minimally adjusted and fully adjusted multiple logistic regression models were used to assess the association between two surgical techniques and PPOI. The covariates were considered in the fully adjusted model if the matched odds ratio changed at least 10 percent as the result of adding those covariates.^19^ Also, stratified and interaction analyses were undertaken according to the two classification schemes of baseline characteristics. Finally, the non‐adjusted, minimally adjusted and fully adjusted multiple regression analyses in the stratified populations established the effect of surgical techniques on PPOI. All of these analyses were performed using the statistical software packages R (http://www.R-project.org, The R Foundation) and EmpowerStates (http://www.empowerstates.com, X & Y Solutions, Inc, Boston, MA). The 95% CIs for between‐group differences were calculated and *P *< .05 (two‐sided) were considered statistically significant.

This study was reported according to the Strengthening the Reporting of Observational Studies in Epidemiology (STROBE) guidelines.^20^

## 3 RESULTS

### 3.1 Patient characteristics

The characteristics of the patients in the open and laparoscopic groups are presented in Table 1. A total number of 162 patients were included in the analysis, with a predominance (n = 99, 61.1%) in the laparoscopic group. The majority of the patients was male (n = 124, 76.5%) and patients in the open group were older than patients in the laparoscopic group. Of the total patients, 32 (19.75%) developed a PPOI. The incidence of PPOI in the open group was higher than the laparoscopic group (28.57% vs 14.14%, *P* = .025). Moreover, the laparoscopic approach was associated with a longer operative time, less intraoperative blood loss, a higher level of postoperative albumin and lower level of postoperative serum potassium than open surgery. Interestingly, opioid analgesic medications were more often prescribed in the postoperative patients with laparoscopic surgery. Otherwise, the distribution of patient characteristics (sex, body mass index, previous abdominal surgery, blood transfusion, tumor stage, postoperative body temperature, postoperative WBC) between both groups was similar.

**Table 1:** The baseline characteristics of patients according to the operation methods

Variable	OS (n = 63)	LS (n = 99)	*P* value
Sex			.499
Female	13 (20.6%)	25 (25.3%)	
Male	50 (79.4%)	74 (74.7%)	
Age (Years)	63.16 ± 10.28	57.19 ± 10.64	**<.001**
BMI (kg/m^2^)	24.59 ± 3.39	24.70 ± 3.41	.833
Previous abdominal surgery			.699
No	50 (79.37%)	81 (81.82%)	
Yes	13 (20.64%)	18 (18.18%)	
Operation time (min)	200.06 ± 51.09	259.54 ± 56.88	**<.001**
Intraoperative blood loss (mL)	253.23 ± 205.03	187.45 ± 144.18	**.018**
Blood transfusion			.106
No	47 (74.60%)	84 (84.85%)	
Yes	16 (25.40%)	15 (15.15%)	
Tumor stage			.900
I‐II	35 (55.56%)	54 (54.55%)	
III‐IV	28 (44.44%)	45 (45.46%)	
Postoperative body temp. (°C)	37.68 ± 0.53	37.53 ± 0.53	.095
Postoperative WBC (×10^9^/L)	12.80 ± 3.27	12.73 ± 3.28	.891
Postoperative albumin (g/L)	31.22 ± 3.13	32.38 ± 3.21	**.025**
Postoperative K^+^ (mmol/L)	3.92 ± 0.44	3.65 ± 0.32	**<.001**
Postoperative opioid analgesic			**.018**
No	46 (73.02%)	54 (54.55%)	
Yes	17 (26.98%)	45 (45.46%)	
PPOI			**.025**
No	45 (71.43%)	85 (85.86%)	
Yes	18 (28.57%)	14 (14.14%)	

Note

Values shown in bold are statistically significant (*P* < .05).

Abbreviations: BMI, body mass index; LS, laparoscopic surgery; OS, open surgery; postoperative body temp., postoperative body temperature; WBC, white blood cell.

### 3.2 The relationship between patient characteristics and PPOI

Univariate analyses were performed to evaluate factors affecting PPOI, and the results are shown in Table 2. Compared to open surgery, the laparoscopic approach was a strong protective factor for the PPOI (OR = 0.41, CI: 0.19‐0.90, *P* = .027). A significantly positive correlation was observed between PPOI and a series of patient characteristics, including: older age (OR = 1.05, CI: 1.01‐1.09, *P* = .009), late surgical pathologic staging (OR = 2.42, CI: 1.09‐5.37, *P* = .030), and postoperative use of opioid analgesic (OR = 3.49, CI: 1.56‐7.81, *P* = .002). Besides, postoperative albumin (OR = 0.83, CI: 0.72‐0.95, *P* = .007) and serum potassium (OR = 0.26, CI: 0.08‐0.81, *P* = .020) were protective indicators of postoperative peristalsis.

**Table 2:** The results of the univariate analysis for factors related to PPOI

Subgroup	Statistics	PPOI	*P* value
Sex			.487
Female	38 (23.46%)	1.0	
Male	124 (76.54%)	0.73 (0.31, 1.76)	
Age (Years)	59.51 ± 10.87	1.05 (1.01, 1.09)	**.009**
BMI (kg/m^2^)	24.66 ± 3.39	0.91 (0.81, 1.02)	.110
Previous abdominal surgery			.574
No	131 (80.86%)	1.0	
Yes	31 (19.14%)	0.74 (0.26, 2.11)	
Operation time (min)	236.41 ± 61.81	0.99 (0.99, 1.00)	.532
Operative blood loss (mL)	229.41 ± 172.75	1.00 (0.99, 1.00)	.693
Blood transfusion			.154
No	131 (80.86%)	1.0	
Yes	31 (19.14%)	1.92 (0.78, 4.71)	
Tumor stage			**.030**
I‐II	89 (54.94%)	1.0	
III‐IV	73 (45.06%)	2.42 (1.09, 5.37)	
Postoperative body temp. (°C)	37.59 ± 0.53	0.99 (0.47, 2.05)	.969
Postoperative WBC (×10^9^/L)	12.76 ± 3.26	1.04 (0.92, 1.17)	.572
Postoperative albumin (g/L）	31.93 ± 3.22	0.83 (0.72, 0.95)	**.007**
Postoperative K^+^ (mmol/L)	3.75 ± 0.40	0.26 (0.08, 0.81)	**.020**
Postoperative opioid analgesic			**.002**
No	100 (61.73%)	1.0	
Yes	62 (38.27%)	3.49 (1.56, 7.81)	
Operation methods			**.027**
Open surgery	63 (38.89%)	1.0	
Laparoscopic surgery	99 (61.11%)	0.41 (0.19, 0.90)	

Note

Data were reported as a mean ± standard deviation for continuous variables and as a patient percentage for categorical variables. Values shown in bold are statistically significant (*P* < .05).

Abbreviations: BMI, body mass index; postoperative body temp., postoperative body temperature; WBC, white blood cell.

### 3.3 Surgical approaches were independently associated with PPOI

To identify the independent effect of surgical approaches on PPOI, we performed multiple regression analyses in different models (Table 3). Similar to the non‐adjusted model, laparoscopy approach was a significant protective factor for PPOI in the minimally adjusted model (OR = 0.40 CI 0.18‐0.86, *P* = .024, adjusted for sex and BMI). We screened six covariates that significantly affected the matched odds ratio (more than 10%^19^) of the non‐adjusted model, including age, intraoperative blood loss, tumor stage, postoperative albumin, postoperative serum potassium, and a postoperative opioid analgesic. In the fully adjusted model (adjusted for sex, BMI and six screened covariates), the result remained significant (OR = 0.17, CI: 0.05‐0.52, *P* = .002).

**Table 3:** The association of PPOI and operation method in different models

Models	Crude model	Model I	Model II
Operation methods			
OS	1	1	1
LS	0.41 (0.19, 0.90) .027	0.40 (0.18, 0.86) .024	0.21 (0.08, 0.59) .003

Note

Data are shown as the format of OR (95% CI) *P* value. Crude model did not adjust other covariants Model I minimally adjusted for sex and BMI; Model II fully adjusted for sex, BMI, age, intraoperative blood loss, tumor stage, postoperative albumin, postoperative serum potassium, and use of a postoperative opioid analgesic.

Abbreviations: LS, Laparoscopic surgery; OS, Open surgery.

### 3.4 Stratified analyses of surgical approaches on PPOI

**Figure 2:**
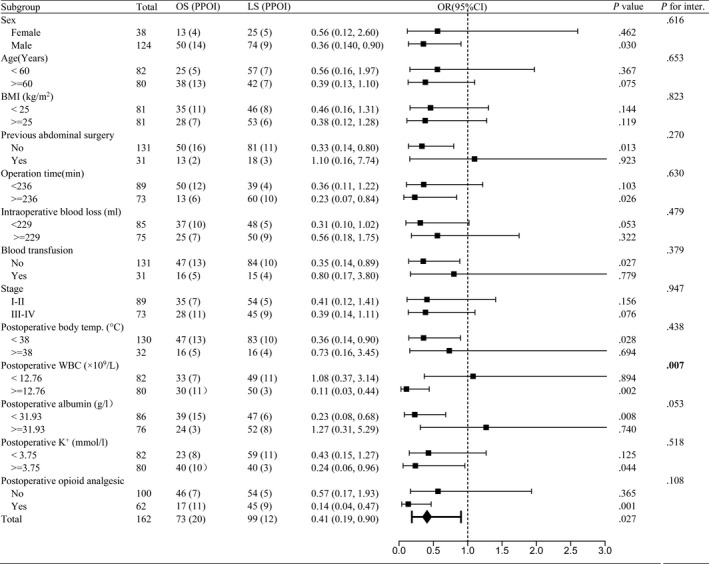
Subgroup analyses of PPOI, according to baseline characteristics. Values shown in bold are statistically significant (*P* < .05). Abbreviations: BMI, body mass index; LS, laparoscopic surgery; OS, open surgery; postoperative body temp., postoperative body temperature; P for inter., P for interaction; WBC, white blood cell

**Figure 3:**
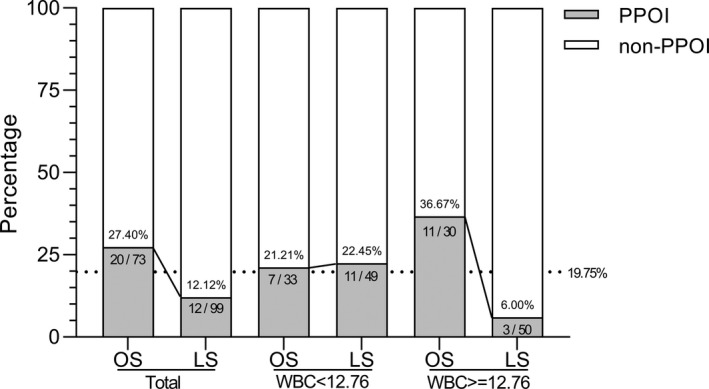
Stacked histogram analyses of PPOI in subgroups of WBC

One way to examine the primary association of interest at different levels of a potential confounding factor is stratified analysis. We further detected the effect of surgical approaches in subgroups (divided by the median or clinical thresholds) of patient characteristics. As was shown in Figure 2, laparoscopy approach had strong protective effects (OR = 0.11, CI:0.03‐0.43, *P* = .001) in the subgroup patients with postoperative WBC counts above 12.76 × 10^9^/L and no significant effects (OR = 1.10, CI: 0.38‐3.23, *P* = .856) in the other subgroup with postoperative WBC counts below 12.76 × 10^9^/L. There was an interaction between surgical approaches and the subgroup of postoperative WBC (*P* for interaction = .006). No significant interactions were found in other subgroup analyses. We further performed stacked histogram analyses to validate the stratified effects vividly. As shown in Figure 3, no significant difference of PPOI was observed between the open and laparoscopic groups in the patients with postoperative WBC counts below 12.76 × 10^9^/L (21.12% vs 22.45%). However, among the patients with postoperative WBC counts above 12.76 × 10^9^/L, PPOI was significantly decreased in patients receiving laparoscopic surgery (6%) than those receiving open surgery (36.67%). Furthermore, we performed multiple regression analysis in different models to validate the stratified effects in two or three subgroups (Table 4). In the two group analysis, laparoscopic surgery had a lower risk of PPOI than open group for the patients with WBC counts above the middle level. The results remained significant in the minimally adjusted model I and the fully adjusted model II. In the three group analysis of WBC count, this effect remained statistically significant in the middle and high tertile counts of WBC and the stratified effect remained stable in the minimally adjusted model I and the fully adjusted model II.

**Table 4:** Stratified analyses of the interaction between operation methods and postoperative WBC in different models

Models	Crude model		Model I		Model II	
Postoperative WBC	OR (95% CI)	*P* value	OR (95% CI)	*P* value	OR (95% CI)	*P* value
WBC (2‐Quantile)						
Q1 (<12.76)	1.08 (0.37, 3.14)	.894	1.03 (0.34, 3.07)	.963	0.37 (0.09, 1.57)	.178
Q2 (≥12.76)	0.11 (0.03, 0.44)	.002	0.07 (0.01, 0.35)	.001	0.04 (0.01, 0.37)	.004
WBC (Tertile)						
T1 (<11.50)	1.74 (0.40, 7.52)	.456	1.66 (0.38, 7.31)	.504	1.00 (0.16, 6.08)	.996
T2 (11.50‐14.10)	0.22 (0.05, 0.96)	.044	0.24 (0.05, 1.04)	.057	0.05 (0.01, 0.63)	.021
T3 (>14.10)	0.16 (0.04, 0.72)	.017	0.05 (0.01, 0.50)	.011	0.01 (0.00, 0.48)	.019

Note

Data are shown as the odds ratio format of the PPOI risk of laparoscopic surgery vs open surgery. Crude model did not adjust other covariants. Model I minimally adjusted for sex and BMI; Model II fully adjusted for sex, BMI, age, intraoperative blood loss, tumor stage, postoperative albumin, postoperative serum potassium, and use of a postoperative opioid analgesic.

Abbreviations: WBC, white blood cell, which omits unit of × 10^9^/L.

## 4 DISCUSSION

There have been numerous reports on PPOI after colectomy with prediction or post hoc analyses.^3^, ^17^, ^18^, ^21^, ^22^ However, little is known about risk factors of PPOI for patients with gastric cancer. This study analyzed 15 potential factors of PPOI after resection of gastric cancer in 162 patients. We identified the following risk factors that had a significantly positive correlation with PPOI: open surgery, older age, late surgical pathologic staging, postoperative use of opioid analgesic, low level of postoperative albumin and serum potassium. Our study confirmed that the laparoscopic approach was a significant protective factor for PPOI after gastric surgery and multivariable analysis was performed to ensure that adjusting simultaneously for multiple covariates would not change the result. Furthermore, stratified analyses demonstrated that laparoscopy approach had strong protective effects in the subgroup patients with high level of postoperative WBC.

In our study, the frequency of PPOI after gastric surgery was 19.75%, which was lower to that reported by Huang et al (32.4%) in gastric cancer^7^ and similar to previous studies about colectomy by Hain et al (15%) and Chapuis et al (14%).^5^, ^6^ The frequency of PPOI was variable in different studies due to the ambiguity about the definition. Controversies have mainly focused on the duration of ileus that should be regarded as prolonged. An observational study of 2400 consecutive patients determined 3 days as prolonged ileus,^5^ whereas Dai et al specified 4 days and Artinyan et al defined more than 6 days.^21^, ^23^ A global survey and systematic review extracted definitions from 52 identified trials and proposed 4 days as a standardized endpoint for PPOI.^2^ It was well accepted in subsequent studies, and we also adopted this definition as diagnostic criteria in our study.^3^, ^16^ Given the variability in the definitions of this significant complication, further research is necessary to establish a more precise, validated definition.

In the present study, we identified the open operative approach as an independent risk factor for PPOI, which was consistent with the results reported in other studies.^14^, ^24^ Laparoscopic surgery has been shown to have similar or better long‐term outcomes compared with open surgery in gastric cancer patients, even in patients with advanced tumor stage.^10^ A meta‐analysis comparing laparoscopic total gastrectomy with open total gastrectomy for the treatment of gastric cancer and laparoscopic surgery was associated with a quicker recovery of gastrointestinal motility.^25^ However, the results of an observational study of 2400 consecutive patients showed that the open vs laparoscopic operation did not have a significant effect in the multivariable model.^5^ Characteristic analyses of this large sample population indicated that open operation was significantly more common among males and at urgent resection, which may displace effect from open vs laparoscopic operation in the multivariable model. Our results showed that laparoscopic surgery took longer operative time to perform but was associated with less intraoperative blood loss, quicker recovery, and better postoperative outcomes. Recovery of gastrointestinal function after abdominal surgery has been shown to occur an average of 2 days later in patients who undergo open vs laparoscopic surgery.^26^ The laparoscopic approach is associated with minimal trauma and lower level of systemic inflammation, which may lead to a faster recovery of postoperative bowel function.^27^, ^28^, ^29^ Our results also demonstrated that the laparoscopic approach had predominance in the subgroup patients with higher risk of infection. In our study, laparoscopic operations were significantly more common among patients postoperatively using opioid analgesics which may inhibit peristalsis in the gut and increase the incidence of PPOI.^30^, ^31^ The variable imbalance of opioid analgesics in the laparoscopic group was likely to contribute to the open group, which made our results more reliable. In addition, lower postoperative potassium level was associated with the development of PPOI in elective colon resections.^18^ Characteristic analyses in our study indicated that the laparoscopic approach was associated with lower level of postoperative serum potassium than open surgery, which was also likely to contribute to the open group. Therefore, laparoscopic gastrectomy should be recommended in patients with gastric cancer to reduce PPOI.

Several studies have demonstrated the prior abdominal surgery about an increased incidence of PPOI after abdominal surgery.^4^, ^15^, ^32^, ^33^ However, no significant difference in PPOI about prior abdominal surgery was found in our study. We analyzed those studies to examine potential causes of heterogeneity. In addition to the difference of populations, the range of prior abdominal surgery was of prime importance. For example, patients who had previous major abdominal surgery other than appendectomy, hysterectomy, cesarean section, or cholecystectomy were also excluded in the study by Kronberg et al^4^ In our study, we did not restrict the range of previous abdominal surgery, which was not comparable.

There are certain limitations to the present study. As this was a retrospective cohort study, not all potential parameters could be detected. Meanwhile, our findings arose from a single surgical group on gastric cancer and may not be generalizable to other institutions or abdominal operations. There may also have some bias, as the selection of surgical approaches was based on the doctor‐patient preoperative communication. Thus, a prospective randomized controlled trial is needed in the subsequent studies.

## 5 CONCLUSION

In agreement with findings from previous studies, results of our study reinforce the view that the type of surgical approach has a relationship with PPOI in patients with gastric cancer, and we suggest that laparoscopic surgery should be recommended in patients if available. Patients who underwent open surgery and were associated with high level of postoperative WBC should be cautious with PPOI, and preventive measures should be considered early.

## CONFLICT OF INTEREST

The authors declare that they have no conflict of interest.

## ETHICAL APPROVAL

All procedures performed in studies involving human participants were in accordance with the ethical standards of our institutional review board and with the 1964 Helsinki Declaration and its later amendments or comparable ethical standards.

## INFORMED CONSENT

Informed consent was obtained from all individual participants included in the study.

## References

[1] Wolthuis AM , Bislenghi G , Lambrecht M , et al. Preoperative risk factors for prolonged postoperative ileus after colorectal resection. Int J Colorectal Dis. 2017;32(6):883‐890. 28444506 10.1007/s00384-017-2824-6

[2] Vather R , Trivedi S , Bissett I . Defining postoperative ileus: results of a systematic review and global survey. J Gastrointest Surg. 2013;17(5):962‐972. 23377782 10.1007/s11605-013-2148-y

[3] Mao H , Milne T , O'Grady G , Vather R , Edlin R , Bissett I . Prolonged postoperative ileus significantly increases the cost of inpatient stay for patients undergoing elective colorectal surgery: results of a multivariate analysis of prospective data at a single institution. Dis Colon Rectum. 2018;62(5):631‐637. 10.1097/DCR.0000000000001301 30543534

[4] Kronberg U , Kiran RP , Soliman M , et al. A characterization of factors determining postoperative ileus after laparoscopic colectomy enables the generation of a novel predictive score. Ann Surg. 2011;253(1):78‐81. 21233608 10.1097/SLA.0b013e3181fcb83e

[5] Chapuis PH , Bokey L , Keshava A , et al. Risk factors for prolonged ileus after resection of colorectal cancer: an observational study of 2400 consecutive patients. Ann Surg. 2013;257(5):909‐915. 23579542 10.1097/SLA.0b013e318268a693

[6] Hain E , Maggiori L , Mongin C , Prost AlDJ , Panis Y . Risk factors for prolonged postoperative ileus after laparoscopic sphincter‐saving total mesorectal excision for rectal cancer: an analysis of 428 consecutive patients. Surg Endosc. 2018;32(1):337‐344. 28656338 10.1007/s00464-017-5681-z

[7] Huang D‐D , Zhuang C‐L , Wang S‐L , et al. Prediction of prolonged postoperative ileus after radical gastrectomy for gastric cancer: a scoring system obtained from a prospective study. Medicine (Baltimore). 2015;94(51):e2242. 26705206 10.1097/MD.0000000000002242 PMC4697972

[8] Chan DC , Liu YC , Chen CJ , et al. Preventing prolonged post‐operative ileus in gastric cancer patients undergoing gastrectomy and intra‐peritoneal chemotherapy. World J Gastroenterol: WJG. 2005;11(31):4776‐4781. 16097043 10.3748/wjg.v11.i31.4776 PMC4398721

[9] Bray F , Ferlay J , Soerjomataram I , Siegel RL , Torre LA , Jemal A . Global cancer statistics 2018: GLOBOCAN estimates of incidence and mortality worldwide for 36 cancers in 185 countries. CA Cancer J Clin. 2018;68(6):394‐424. 30207593 10.3322/caac.21492

[10] Kim H‐H , Han S‐U , Kim M‐C , et al. Long‐term results of laparoscopic gastrectomy for gastric cancer: a large‐scale case‐control and case‐matched Korean multicenter study. J Clin Oncol. 2014;32(7):627‐633. 24470012 10.1200/JCO.2013.48.8551

[11] Hu Y , Huang C , Sun Y , et al. Morbidity and mortality of laparoscopic versus open D2 distal gastrectomy for advanced gastric cancer: a randomized controlled trial. J Clin Oncol. 2016;34(12):1350‐1357. 26903580 10.1200/JCO.2015.63.7215

[12] Yu J , Huang C , Sun Y , et al. Effect of laparoscopic vs open distal gastrectomy on 3‐year disease‐free survival in patients with locally advanced gastric cancer: the CLASS‐01 randomized clinical trial. JAMA. 2019;321(20):1983‐1992. 31135850 10.1001/jama.2019.5359 PMC6547120

[13] Scotland H , Widmer JD , Wildi S , Bueter M , Weber M , Muller MK . How to cope with insufficient pneumoperitoneum and exposure when performing laparoscopic gastric bypass surgery. Langenbecks Arch Surg. 2016;401(3):299‐305. 26887905 10.1007/s00423-016-1379-2

[14] Moghadamyeghaneh Z , Hwang GS , Hanna MH , et al. Risk factors for prolonged ileus following colon surgery. Surg Endosc. 2016;30(2):603‐609. 26017914 10.1007/s00464-015-4247-1

[15] Sugawara K , Kawaguchi Y , Nomura Y , et al. Perioperative factors predicting prolonged postoperative ileus after major abdominal surgery. J Gastrointest Surg. 2018;22(3):508‐515. 29119528 10.1007/s11605-017-3622-8

[16] Vather R , Josephson R , Jaung R , Kahokehr A , Sammour T , Bissett I . Gastrografin in prolonged postoperative ileus: a double‐blinded randomized controlled trial. Ann Surg. 2015;262(1):23‐30. 25575258 10.1097/SLA.0000000000001062

[17] Wolthuis AM , Bislenghi G , Fieuws S , de Buck van Overstraeten A , Boeckxstaens G , D'Hoore A . Incidence of prolonged postoperative ileus after colorectal surgery: a systematic review and meta‐analysis. Colorectal Dis. 2016;18(1):O1‐9. 26558477 10.1111/codi.13210

[18] Kuruba R , Fayard N , Snyder D . Epidural analgesia and laparoscopic technique do not reduce incidence of prolonged ileus in elective colon resections. Am J Surg. 2012;204(5):613‐618. 22906251 10.1016/j.amjsurg.2012.07.011

[19] Kernan WN , Viscoli CM , Brass LM , et al. Phenylpropanolamine and the risk of hemorrhagic stroke. N Engl J Med. 2000;343(25):1826‐1832. 11117973 10.1056/NEJM200012213432501

[20] von Elm E , Altman DG , Egger M , et al. The Strengthening the Reporting of Observational Studies in Epidemiology (STROBE) statement: guidelines for reporting observational studies. Lancet. 2007;370(9596):1453‐1457. 18064739 10.1016/S0140-6736(07)61602-X

[21] Dai X , Ge X , Yang J , et al. Increased incidence of prolonged ileus after colectomy for inflammatory bowel diseases under ERAS protocol: a cohort analysis. J Surg Res. 2016;212:86‐93. 28550927 10.1016/j.jss.2016.12.031

[22] Juárez‐Parra MA , Carmona‐Cantú J , González‐Cano JR , Arana‐Garza S , Treviño‐Frutos RJ . Risk factors associated with prolonged postoperative ileus after elective colon resection. Revista de Gastroenterología de México (English Edition). 2015;80(4):260‐266. 10.1016/j.rgmx.2015.08.002 26601818

[23] Artinyan A , Nunoo‐Mensah JW , Balasubramaniam S , et al. Prolonged postoperative ileus‐definition, risk factors, and predictors after surgery. World J Surg. 2008;32(7):1495‐1500. 18305994 10.1007/s00268-008-9491-2

[24] Jiang L , Yang K‐H , Guan Q‐L , et al. Laparoscopy‐assisted gastrectomy versus open gastrectomy for resectable gastric cancer: an update meta‐analysis based on randomized controlled trials. Surg Endosc. 2013;27(7):2466‐2480. 23361259 10.1007/s00464-012-2758-6

[25] Xiong JJ , Nunes QM , Huang W , et al. Laparoscopic vs open total gastrectomy for gastric cancer: a meta‐analysis. World J Gastroenterol: WJG. 2013;19(44):8114‐8132. 24307808 10.3748/wjg.v19.i44.8114 PMC3848162

[26] Vather R , Josephson R , Jaung R , Robertson J , Bissett I . Development of a risk stratification system for the occurrence of prolonged postoperative ileus after colorectal surgery: a prospective risk factor analysis. Surgery. 2015;157(4):764‐773. 25724094 10.1016/j.surg.2014.12.005

[27] Wehner S , Vilz TO , Stoffels B , Kalff JC . Immune mediators of postoperative ileus. Langenbecks Arch Surg. 2012;397(4):591‐601. 22382699 10.1007/s00423-012-0915-y

[28] Baig MK , Wexner SD . Postoperative ileus: a review. Dis Colon Rectum. 2004;47(4):516‐526. 14978625 10.1007/s10350-003-0067-9

[29] Kehlet H , Holte K . Review of postoperative ileus. Am J Surg. 2001;182(5A Suppl):3S‐10S. 11755891 10.1016/s0002-9610(01)00781-4

[30] Koo KC , Yoon YE , Chung BH , Hong SJ , Rha KH . Analgesic opioid dose is an important indicator of postoperative ileus following radical cystectomy with ileal conduit: experience in the robotic surgery era. Yonsei Med J. 2014;55(5):1359‐1365. 25048497 10.3349/ymj.2014.55.5.1359 PMC4108824

[31] Becker G , Blum HE . Novel opioid antagonists for opioid‐induced bowel dysfunction and postoperative ileus. The Lancet. 2009;373(9670):1198‐1206. 10.1016/S0140-6736(09)60139-2 19217656

[32] Kim IK , Kang J , Baik SH , Lee KY , Kim NK , Sohn SK . Impact of prior abdominal surgery on postoperative prolonged ileus after ileostomy repair. Asian J Surg. 2016;41(1):86‐91. 27542335 10.1016/j.asjsur.2016.07.006

[33] Yamamoto M , Okuda J , Tanaka K , et al. Effect of previous abdominal surgery on outcomes following laparoscopic colorectal surgery. Dis Colon Rectum. 2013;56(3):336‐342. 23392148 10.1097/DCR.0b013e31827ba103

